# Predicting Spatial Patterns of Sindbis Virus (SINV) Infection Risk in Finland Using Vector, Host and Environmental Data

**DOI:** 10.3390/ijerph18137064

**Published:** 2021-07-01

**Authors:** Ruut Uusitalo, Mika Siljander, C. Lorna Culverwell, Guy Hendrickx, Andreas Lindén, Timothée Dub, Juha Aalto, Jussi Sane, Cedric Marsboom, Maija T. Suvanto, Andrea Vajda, Hilppa Gregow, Essi M. Korhonen, Eili Huhtamo, Petri Pellikka, Olli Vapalahti

**Affiliations:** 1 Department of Geosciences and Geography, University of Helsinki, P.O. Box 64, FI-00014 Helsinki, Finland; mika.siljander@helsinki.fi (M.S.); juha.aalto@helsinki.fi (J.A.); petri.pellikka@helsinki.fi (P.P.); 2 Department of Virology, University of Helsinki, Haartmaninkatu 3, P.O. Box 21, FI-00014 Helsinki, Finland; lorna.culverwell@helsinki.fi (C.L.C.); maija.t.suvanto@helsinki.fi (M.T.S.); essi.m.korhonen@helsinki.fi (E.M.K.); eili.huhtamo@helsinki.fi (E.H.); olli.vapalahti@helsinki.fi (O.V.); 3 Department of Veterinary Biosciences, University of Helsinki, Agnes Sjöberginkatu 2, P.O. Box 66, FI-00014 Helsinki, Finland; 4 Department of Life Sciences, Natural History Museum, Cromwell Road, London SW5 7BD, UK; 5 AVIA-GIS, Risschotlei 33, 2980 Zoersel, Belgium; ghendrickx@avia-gis.com (G.H.); cmarsboom@avia-gis.com (C.M.); 6 Natural Resources Institute Finland (LUKE), P.O. Box 2, FI-00791 Helsinki, Finland; andreas.linden@luke.fi; 7 Department of Health Security, Finnish Institute for Health and Welfare, P.O. Box 30, FI-00271 Helsinki, Finland; timothee.dub@thl.fi (T.D.); jussi.sane@thl.fi (J.S.); 8 Finnish Meteorological Institute, P.O. Box 503, FI-00101 Helsinki, Finland; Andrea.Vajda@fmi.fi (A.V.); hilppa.gregow@fmi.fi (H.G.); 9 Helsinki Institute of Sustainability Science, University of Helsinki, P.O. Box 4, FI-00014 Helsinki, Finland; 10 Institute for Atmospheric and Earth System Research, University of Helsinki, P.O. Box 64, FI-00014 Helsinki, Finland; 11 Virology and Immunology, Diagnostic Center, HUSLAB, Helsinki University Hospital, P.O. Box 400, FI-00029 Helsinki, Finland

**Keywords:** Pogosta disease, vector-borne disease, Sindbis virus infection, mosquitoes, predictive mapping, disease modelling

## Abstract

Pogosta disease is a mosquito-borne infection, caused by Sindbis virus (SINV), which causes epidemics of febrile rash and arthritis in Northern Europe and South Africa. Resident grouse and migratory birds play a significant role as amplifying hosts and various mosquito species, including *Aedes cinereus*, *Culex pipiens*, *Cx. torrentium* and *Culiseta morsitans* are documented vectors. As specific treatments are not available for SINV infections, and joint symptoms may persist, the public health burden is considerable in endemic areas. To predict the environmental suitability for SINV infections in Finland, we applied a suite of geospatial and statistical modeling techniques to disease occurrence data. Using an ensemble approach, we first produced environmental suitability maps for potential SINV vectors in Finland. These suitability maps were then combined with grouse densities and environmental data to identify the influential determinants for SINV infections and to predict the risk of Pogosta disease in Finnish municipalities. Our predictions suggest that both the environmental suitability for vectors and the high risk of Pogosta disease are focused in geographically restricted areas. This provides evidence that the presence of both SINV vector species and grouse densities can predict the occurrence of the disease. The results support material for public-health officials when determining area-specific recommendations and deliver information to health care personnel to raise awareness of the disease among physicians.

## 1. Introduction

Mosquito-borne viruses are responsible for many notable human diseases worldwide and their transmission is a result of complex interactions between climate, vectors, vertebrate hosts and human behaviour [1,2]. Spatial analysis and statistical modeling approaches are commonly used to understand these interactions and their implications for the spread of vector-borne diseases [3,4,5,6]. While high and low temperature extremes are increasing, winter, in particular, may offer favourable conditions for the spread of exotic vector-borne diseases. Endemic mosquito-borne pathogens including Sindbis virus (SINV) are already circulating in northern Europe [7]. Sindbis virus circulates within an enzootic transmission cycle involving mosquito vectors and avian hosts, but it is transmitted to humans via bridge vector species in epidemic transmission events [8]. Spatial modeling approaches can predict vector distributions and infectious disease risks, and therefore may provide strategies for more efficient resource allocation regarding targeted surveillance, prevention, and control [9,10].

Mosquito-transmitted SINV is a member of the Western equine encephalitis antigenic complex of alphaviruses that was originally isolated from *Culex pipiens* Linneaus and/or *Culex univittatus* Theobald collected in Sindbis village, Nile Delta (Egypt) in 1952 [11,12]. Pogosta disease also known as Sindbis fever, Ockelbo disease in Sweden, and Karelian fever in Russian Karelia, is the result of SINV infection in humans. It manifests with fever, rash, headache, myalgia, arthralgia, nausea, conjunctivitis and pharyngitis. After the acute phase, long-lasting joint pain and tendon insertions occur in 25% of infected individuals [13,14,15,16]. The incubation period for SINV is 5–7 days before the onset of symptoms with IgM and IgG antibodies are detectable within 8 and 11 days since onset, respectively, resulting a time lag of 2–3 weeks from transmission to serological diagnosis [15,17]. As no vaccine or specific etiologic treatment is available, clinical care is strictly symptomatic [17]. Infections are notifiable in Australia and some European, Asian and African countries [18], but despite widespread circulation of SINV in Australasia and Africa, human outbreaks are associated only with SINV genotype I (SINV-I), and have only been documented in Northern Europe and South Africa [19,20,21]. In Finland, tularemia and Pogosta disease are the two most common mosquito-borne diseases [22]. Pogosta disease cases are recorded annually, and the estimated seroprevalence of SINV was 5.2% between 1995–2003. A disease epidemic has been seen approximately every seven years, where hundreds or even thousands of patients are infected, until 2009, when an anticipated outbreak did not occur [23,24]. The majority of clinical cases in Northern Europe occur in August and September [25], coinciding with the temporal distribution of the bridge vectors *Aedes cinereus* Meigen, and *Culex pipiens* Linnaeus [8]. However, there is no national public health surveillance for SINV in mosquitoes in Finland. *Aedes cinereus* and *Culex pipiens* are abundant during the late summer and early autumn in Finland, a time when the relative humidity increases throughout the country.

Sindbis virus mainly circulates in enzootic cycles between birds, which act as amplifying hosts, including both resident grouse (Galliformes) and migratory birds, mainly passerines, such as thrushes (Turdidae) [21,23,26]. Ornithophilic mosquitoes, *Culex pipiens*, *Cx. torrentium* Martini and *Culiseta morsitans* Theobald maintain the virus in these cycles [26,27]. In addition, *Aedes cinereus* and *Culex pipiens* are bridge vectors, which transmit the virus from birds to humans [13,28,29,30]. In recent studies, SINV-I has also been detected in or isolated from *Ochlerotatus* species [31,32,33], which would implicate their potential as additional bridge vectors. Females, which are zoophilic and anthropophilic, are morphologically identical to *Ae. geminus* Peus, whose role in transmission cycles is poorly understood. While both *Cx. pipiens* and *Cx. torrentium* are ornithophilic, *Cx. pipiens* is also known to occasionally bite humans. In contrast, *Cx. torrentium* has been reported to not bite humans, even in a laboratory setting [34]. *Culiseta morsitans* adults are ornithophilic, but have occasionally been observed to bite reptiles, small mammals and humans [34]. The overwintering mechanisms of SINV in bird hosts are largely unknown. Most of the studies have detected SINV in mosquitoes, but some experimental vector-competence work has been done on *Culex pipiens* and *Cx. torrentium* [35]. Although a mosquito could have picked up the virus in its blood meal, and the virus from that blood meal could be detected, this is usually considered in virus studies and the blood-fed individuals are sorted separately from the rest of the specimens for processing and analysis. In Sweden, SINV has also been detected in hibernating, on-blood fed *Cx. pipiens* mosquitoes, suggesting that the virus also overwinters in this vector species, which may be important mechanism for virus survival and persistence in nature [36].

**Figure 1:**
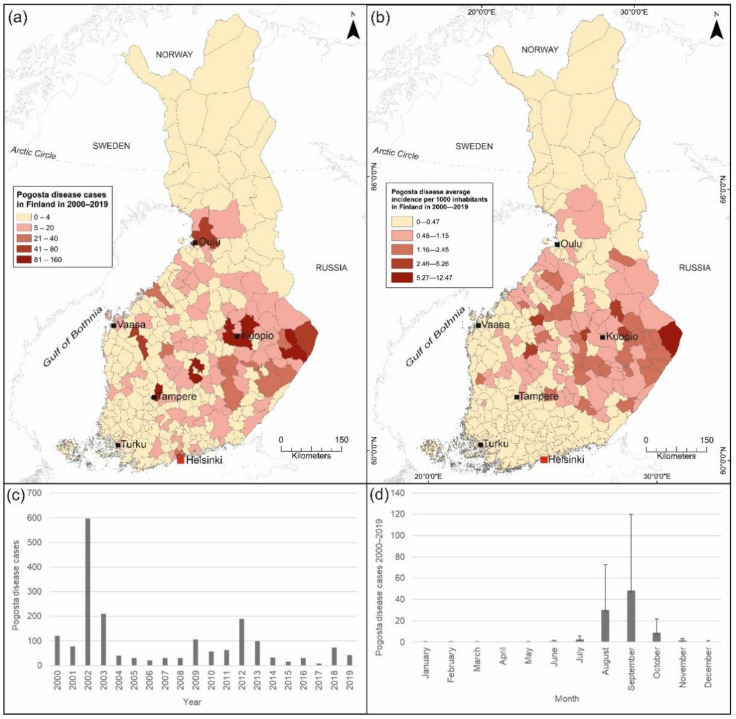
(**a**) Human Pogosta disease cases registered in Finland during 2000–2019. (**b**) The average incidence of Pogosta disease per 1000 inhabitants over a 20-year period in Finland. (**c**) Annual number of Pogosta disease cases, and (**d**) mean monthly Pogosta disease cases notified in Finland in 2000–2019 [22]. An incidence rate below 0.48 was considered as absence of the disease.

Climate plays an important role in the transmission of vector-borne diseases, since arthropods, including mosquitoes, are sensitive to changes in environmental conditions. Weather, climate change and the environment influence the habitat suitability, vector activity and the rate of vector development [13,37,38,39]. The replication of pathogens within vectors occurs faster at warm temperatures [40]. Temperature and precipitation patterns also influence vector densities [41,42]. Generally, warm temperatures and increased rainfall positively affect vector densities but extreme high temperatures combined with decreased rainfall may reduce mosquito populations [43]. The duration of vector development is also influenced by the thickness of snow cover, especially in the spring [44]. Outbreaks of Pogosta disease have been strongly concentrated in primarily eastern and central regions in Finland with dense forest cover and abundant lakes (Figure 1a), implying a good potential for predicting and understanding the drivers of the observed spatial pattern. In an earlier study, snow depth, air temperature in May–July and the proportion of regulated lakes have been found to influence the number of SINV infections [21,45]. Despite these observations, the presence or abundance of known vector and host species have not been studied to determine their effects on the risk of human Pogosta disease infections in Finland.

In this study, we apply spatial analysis—in particular geographic information system (GIS) and species distribution modeling (SDM) techniques—to better understand how biotic and environmental drivers contribute to the distinct distribution of the Pogosta disease risk in Finland. More specifically, the objectives of this study were to (1) predict the environmental suitability and spatial distribution of vectors known to transmit SINV, (2) to use the resulting predictions together with host and environmental data to estimate the risk of Pogosta disease across Finland, and (3) to identify the most influential predictors driving the spatial patterns of this risk.

## 2. Materials and Methods

### 2.1. Pogosta Disease Data

Finland (59°50′ N, 20°38′ E, 70°09′ N, 31°30′ E), located in Northern Europe between Sweden and Russia (Figure 1a,b), is subdivided at various administrative levels following the Nomenclature of Territorial Units for Statistics (NUTS) system. Patient data was obtained from the National Infectious Diseases Register [22], which included serologically confirmed Pogosta disease cases (*n* = 1825) by municipality of residence from 2000–2019 (Figure 1a). Data on laboratory-confirmed SINV infections is collected routinely through the NIDR. By law, Finnish laboratories are expected to notify findings of a number of microbes specified in the Finnish Communicable Disease Act and Decree, including Sindbis virus infection. A laboratory notification contains the following: identification information, place of treatment, place of residence, specimen collection date, findings, laboratory method, and reporting laboratory [22].

An average of 91 cases were reported annually (varying from 8 to 597) with an incidence of 1.7/100,000. We calculated the incidences for each municipality per 1000 inhabitants between 2000–2019 and calculated the average incidence of all municipalities (0.48/1000) over a 20-year period (Figure 1b). Municipalities with incidence rates above 0.48 were set as a threshold for ‘presence’ municipalities (*n* = 97), and the rest were considered as ‘absence’ municipalities (*n* = 213; Figure 1b).

While cases of Pogosta disease were detected annually, outbreaks, defined in this study as annual occurrence of over 100 cases, were reported in 2000, 2002, 2003, 2009, 2012 and 2013 (Figure 1c). Although no outbreaks occurred after 2013, 72 cases were registered in 2018. Most diagnoses were made and notified in September (on average 48 annual cases over the study period), but many were also notified in August and October (30 and 9 on average, respectively; Figure 1d). During the winter and summer months (excluding August), the number of cases remained low.

### 2.2. Data of Potential SINV Vectors

Mosquito presence data were collected in Finland in 2009 [46], and presence/absence data between 2012–2018 [47]; these were combined for potential SINV vectors. Due to the lack of reliable identification methods to distinguish adult females of *Cx. pipiens* from *Cx. torrentium*, and *Ae. cinereus* from *Ae. geminus*, data were combined to *Cx. pipiens/torrentium* and *Ae. cinereus/geminus*. Presence data were considered as the actual locations of a given species, and absence data were randomly selected from the more than 900 possible locations where collections were made but the species of interest to this study were not found. Altogether, there were 116 presence locations for *Cs. morsitans*, 144 for *Cx. pipiens/torrentium* and 180 for *Ae. cinereus/geminus*. The number of absences was equally weighted to the presences as recommended to build reliable species distribution models [48].

### 2.3. SINV Host Species Data

As SINV is known to circulate in both resident Galliformes and migratory birds, we employed grouse abundance data from the Wildlife Triangle Census, coordinated by the Natural Resources Institute Finland (LUKE). Birds are monitored by voluntary hunters in late July and early August along 12 km–long triangle-shaped line transects [49]. The transects are walked in three-man chains, with the aim to flush all birds from a 60 m wide belt, enabling the calculation of absolute density estimates (individuals/km^2^). Grouse data were compiled by first calculating the average annual densities of willow grouse (*Lagopus lagopus* Linnaeus), black grouse (*Lyrurus tetrix* Linnaeus), capercaillie (*Tetrao urogallus* Linnaeus) and hazel grouse (*Tetrastes bonasia* Linnaeus) in Finnish municipalities between 2000–2019. For each municipality, all triangles within a 100-km radius from the geographical center of the municipality were included. Annual average densities were further averaged across all years to create one average figure per municipality, for the spatial analysis.

### 2.4. Environmental and Other Predictors

Environmental and other predictors were selected based on factors which are known to influence the distributions of vectors and SINV infections [8,21,23,34]. Environmental data for Finland were obtained from various sources and included interpolated data, data directly obtained from satellite imagery or data derived from GIS layers or satellite imagery. Details of the predictor data are provided in Table 1. Altogether, the vector dataset included 31 predictors, and the Pogosta disease dataset included 33 predictors before running a multicollinearity analysis (Section 2.5).

### 2.5. Data Analysis

We used the biomod2 platform in R [60] and VECMAP software to create species distribution models in order to identify areas with suitable habitat conditions for potential SINV vectors and human SINV infections [61,62,63]. All geospatial datasets, including environmental and other data, were processed in ESRI ArcGIS (version 10.3.1) (ESRI, Redlands, CA, USA), and were set to the same spatial extent, geographic coordinate system (EUREF FIN TM35FIN, epsg:3067) and resolution (1 km × 1 km). To model vector distributions, the dataset comprised potential vectors’ presence/absence data, and climatic and environmental predictors. The dataset compiled to model for Pogosta disease included the presence/absence data of Pogosta disease by municipality, and outputs of the vector models, host density data, and environmental data. As Pogosta disease data was obtained per municipality, the zonal mean values of predictor data per municipality were calculated. Multicollinearity of the variables was investigated using Variance Inflation Factors (VIFs) as implemented in R package usdm [64,65]. The VIFs of the predictors were calculated and correlated variables were excluded in a stepwise procedure using a commonly applied threshold value of 5 [66]. The resulting dataset included 21 predictors in the SINV vector modeling (Table 2a), and 19 predictors in the Pogosta disease modeling (Table 2b).

**Figure 2:**
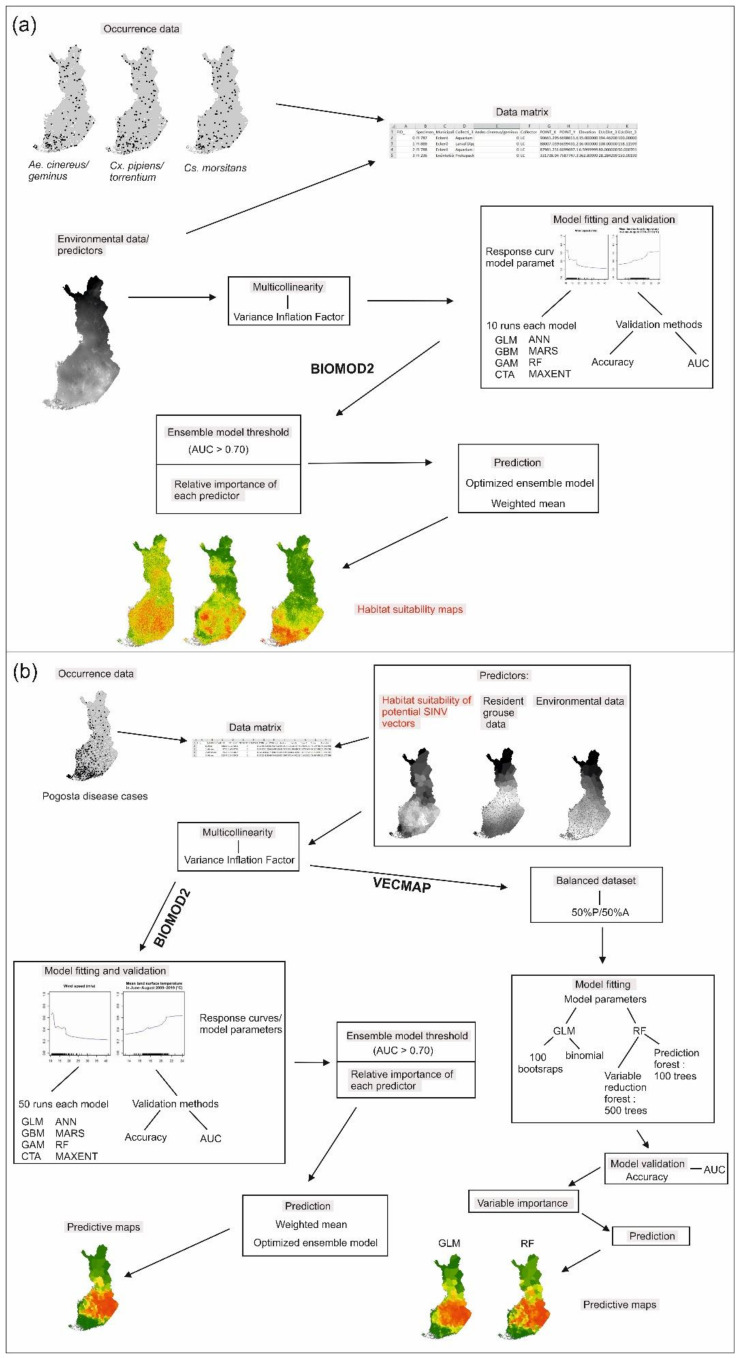
Workflow of modeling the spatial distribution of (**a**) potential SINV vectors with biomod2 approach, and (**b**) Pogosta disease with biomod2 and VECMAP approach.

The workflow to analyse (a) potential SINV vectors and (b) Pogosta disease is presented in Figure 2a,b. We first applied the ensemble approach, which combines predictions across different modeling methods, in the biomod2 package (version 3.4.6) [62] in R to model the distribution of SINV vectors and Pogosta disease risk in Finland. The following eight predictive modeling techniques were employed: generalized linear models (GLM) [67], generalized additive models (GAM) [68], classification tree analysis (CTA) [69], artificial neural networks (ANN) [70], multivariate adaptive regression splines (MARS) [71], generalized boosting models (GBM) [72], random forest (RF) [73], and maximum entropy (MAXENT) [74]. Flexible discriminant analysis (FDA) and surface range envelope (SRE) were excluded due to generally poor predictive performance [75,76,77]. Models were mostly run using the default settings of biomod2 with the following exception: we used the function GAM in mgcv package, with k = 3 as the basis dimension for the thin plate smoothing terms [78]. We used a cross-validation technique where we split the dataset into two subsets, one to calibrate the models (70%) and another to evaluate the models (30%). We repeated the calibration and evaluation sets 10 times (80 model evaluation runs in total) for vector modeling, and 50 times (400 model evaluation runs in total) for Pogosta disease modeling [79]. The area under the receiver operating characteristic (AUC) value was used to assess the model performance in the analyses; scores range from 0 to 1, with 0.5 being the threshold for predictions better than random [80,81]. Sensitivity (the proportion of observed presences) and specificity (the proportion of observed absences) were calculated to quantify the omission errors [80]. Standardized values for relative contribution of the predictors were extracted from the biomod2 output and compared to assess the most powerful variables. Partial dependency plots were generated to show the predictors’ estimated effects on the species and disease distributions. To reduce the uncertainty related to the choice of a single modeling technique, we built ensemble predictions using the weighted mean method. This approach produces the ensemble prediction by averaging predictions across the best-performing individual models (0.7 < AUC < 1.0) and weights them based on their cross-validation performance. Predictions based on weighted mean ensemble models were used as an input for habitat suitability maps of SINV vectors and the Pogosta disease risk map.

Second, we used VECMAP (version 2.2.2.4503) [63] software in order to test the consistency of the results. In VECMAP, we used GLM and RF models to estimate the disease risk. GLM and RF models were processed using the default settings of VECMAP. In the GLM model, 100 repetitions of bootstrap resampling were run for both presence and absence datasets. The top 10 ranked variables were selected based on the best performing model number in terms of the Akaike information criterion (AIC). In the RF model, variable reduction forest was run with 500 trees and prediction forest with 100 trees. Variable contribution in RF was measured with a mean decrease in accuracy and a mean decrease in Gini. The model performance was assessed as described above when using the biomod2 package. Prediction maps were first created by using R or VECMAP, and afterwards modified in ArcGIS.

## 3. Results

### 3.1. Modeling of SINV Mosquito Vector Distributions

#### 3.1.1. Predictive Performance

From 80 model runs for *Ae. cinereus/geminus*, the GAM, GBM, MARS and RF models provided AUC values higher than the reliability threshold 0.70, but below 0.75, and comprised the final ensemble model. Similarly, for *Cx. pipiens/torrentium*, the GAM, GBM, CTA, MARS and RF models resulted in AUC values above 0.70, but below 0.78. All models resulted in high AUC values (0.71–0.90) for *Cs. morsitans* suggesting fair to good predictive power. Sensitivity and specificity rates (by AUC) for estimating the distribution of potential vectors based on weighted mean ensemble model resulted in rates above 85.0%. To estimate *Ae. cinereus/geminus* and *Cs. morsitans* distributions, a better ability to identify suitable environments (sensitivity 93.4% and 96.6%) than unsuitable environments (specificity 88.6% and 87.5%) was presented. In contrast, when estimating *Cx. pipiens/torrentium* distributions, the ensemble model better identified unsuitable environments (sensitivity = 89.5%, specificity = 93.3%).

#### 3.1.2. Predictor Contributions to the Distribution of SINV Vectors

**Figure A1:**
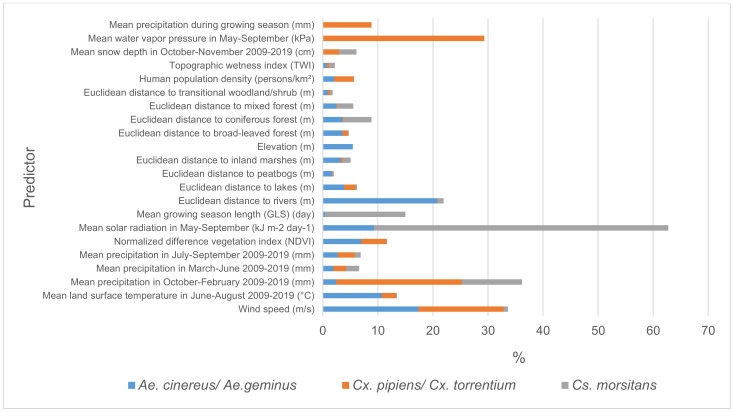
Standardized values for relative contribution of predictors (%) used to predict the distribution of potential SINV vectors in 2000–2019 in Finland. Bars represent the mean value of relative contribution obtained from weighted mean ensemble model.

The relative contribution of influential predictors (%) based on the weighted mean ensemble model varied between mosquito species (Figure A1 in Appendix A). The predictions for *Ae. cinereus/geminus* with the highest relative contribution were the Euclidean distance to river (21%), wind speed (17%), mean land surface temperature in June–August (11%), mean solar radiation in May–September (9%) and normalized difference vegetation index (NDVI) (7%). For *Cx. pipiens/torrentium*, mean water vapor pressure (29%), mean precipitation in October–February (23%), wind speed (15%), mean precipitation during growing season (9%), NDVI (5%) and human population density (4%) were the most important predictors. Mean solar radiation in May–September (53%), mean growing season length (GLS) (15%), mean precipitation in October–February (11%) and the Euclidean distance to coniferous forest (5%) had the highest contribution when predicting *Cs. morsitans* distributions.

**Figure A2:**
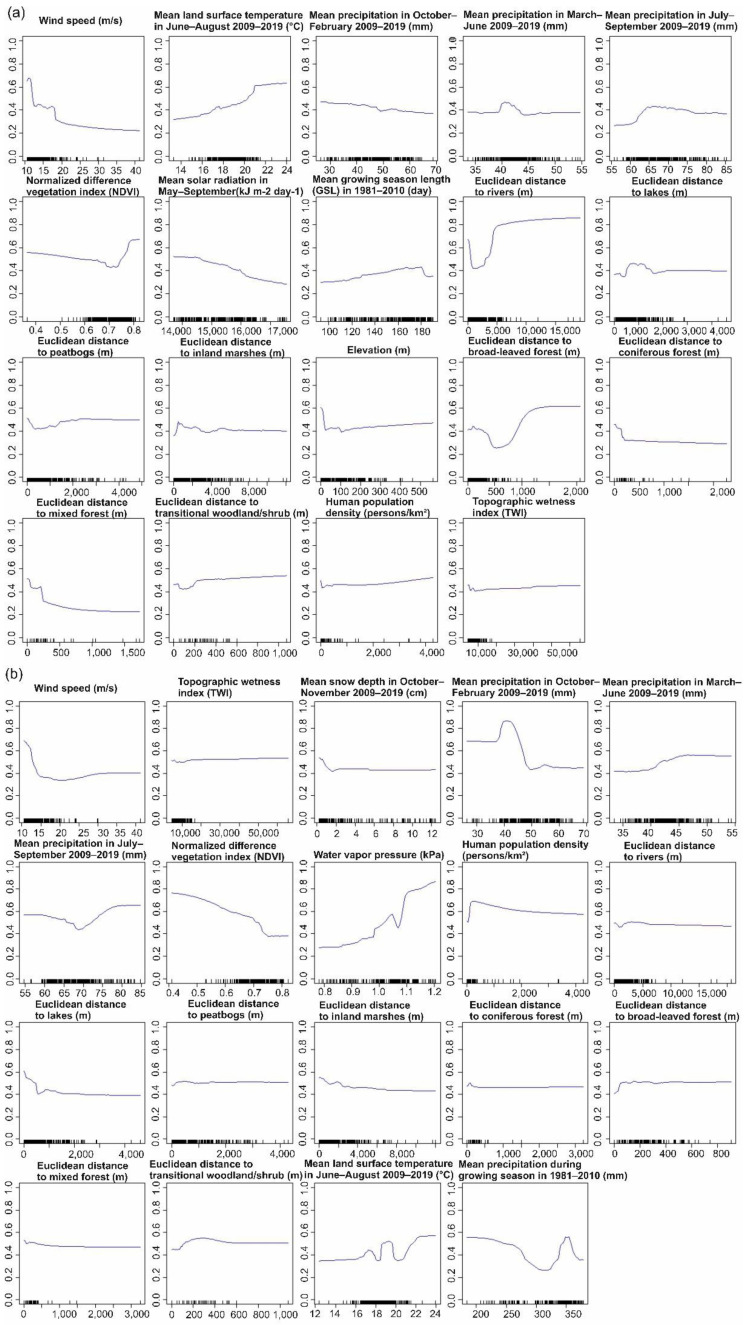

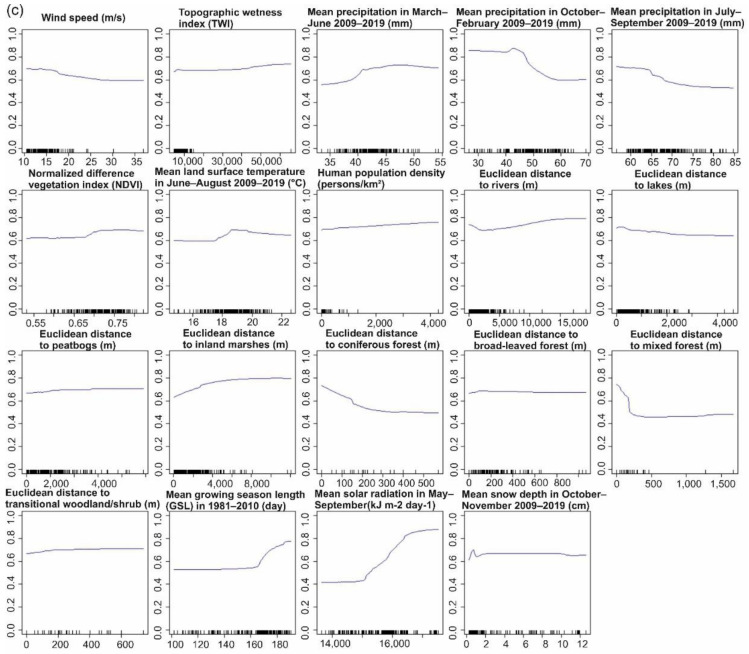
The partial dependency plots for (**a**) *Ae. cinereus/geminus*, (**b**) *Cx. pipiens/torrentium*, and (**c**) *Cs. morsitans* based on average mean ensemble models.

Partial dependency plots for each vector species are shown in Figure A2a–c. High mean temperatures in June–August during 2000–2019, high NDVI and a long growing season in a municipality indicated high probability of *Ae. cinereus/geminus* presence (Figure A2a). Low wind speed, low solar radiation in May–September and short distances to coniferous and mixed forest in the locations were associated with the high probability of an occurrence of *Ae. cinereus/geminus* (Figure A2a). High water vapor pressure and high land surface temperatures in June–August were positively correlated with the probability of *Cx. pipiens/torrentium* occurrence (Figure A2b). The probability of *Cx. pipiens/torrentium* occurrence was also high in locations with low wind speed and sparse vegetation. A long growing season, high precipitation in March–June and high solar radiation in May–September positively influenced *Cs. morsitans* presence (Figure A2c). However, high mean precipitation in July–September and October–February, long distances to coniferous forests and mixed forests indicated a lower probability for *Cs. morsitans* to occur.

#### 3.1.3. Prediction Maps for SINV Vectors

**Figure 3:**
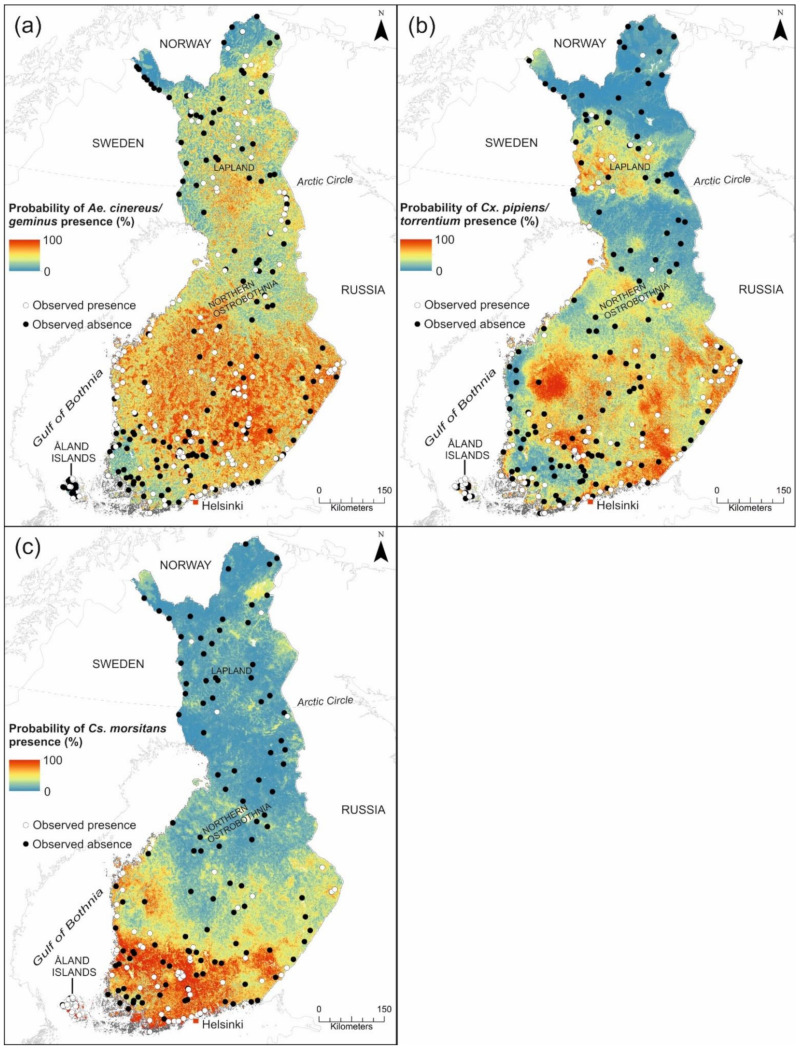
Prediction maps for (**a**) *Ae. cinereus/geminus*, (**b**) *Cx. pipiens/torrentium*, and (**c**) *Cs. morsitans* in Finland based on weighted mean ensemble model.

**Figure 4:**
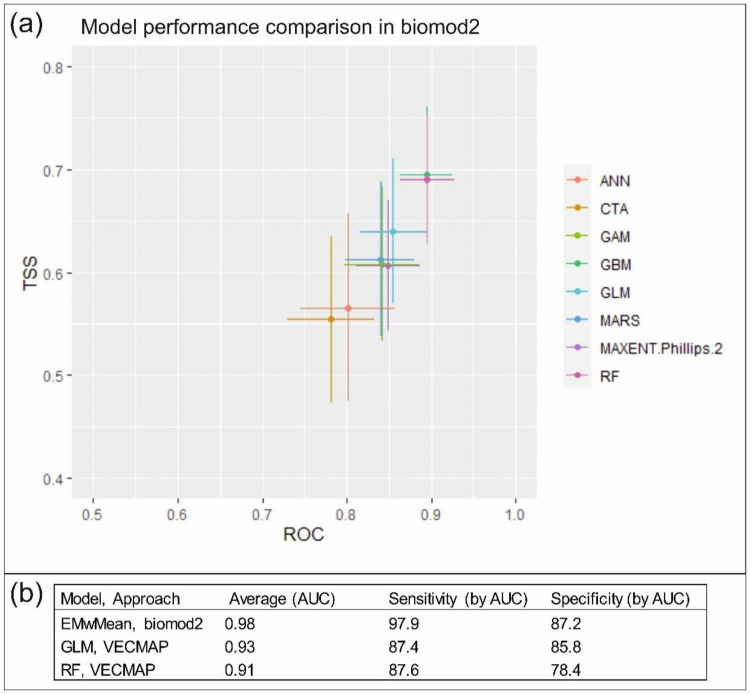
(**a**) Model performance comparison of 8 model algorithms by area under the receiver operating characteristic curve (AUC) and true skill statistics (TSS) values of 50 model runs in biomod2. The points represent the mean values and the solid lines represent the 95% range of variation. ANN = artificial neural networks; CTA = classification tree analysis; GAM = generalized additive models; GBM = generalized boosted models; GLM = generalized linear models; MARS = multivariate additive regression splines; MAXENT = maximum entropy models; RF = random forest model. (**b**) Model performance based on weighted mean ensemble model (EMwMean) in biomod2 and GLM and RF models in VECMAP.

Suitability maps for potential SINV vectors are shown in Figure 3a–c. In this study, low probability of presence/risk is interpreted as 0–30%, moderate probability/risk as 31–60%, and high probability/risk as 61–100%. The areas with high probability for *Ae. cinereus/geminus* to occur were located in central, eastern and western Finland (Figure 4a). The probability of *Ae. cinereus/geminus* presence was also high in Lapland, excluding the northernmost Lapland (0–30%). The areas with moderate probability of *Ae. cinereus/geminus* presence were predicted to occur throughout Finland (30–70%). Southwestern Finland, including the majority of the Åland Islands, was predicted to have low probability for *Ae. cinereus/geminus* presence. High probability for *Cx. pipiens/torrentium* presence was found in central Lapland and most of southern and central Finland, including the Åland Islands and the coastal areas (Figure 3b). In contrast, eastern Northern Ostrobothnia, southern and northern Lapland, and a narrow area in western Finland, were estimated to have a low probability for *Cx. pipiens/torrentium* occurrence. High suitability for *Cs. morsitans* was estimated across southern Finland, including coastal areas, the Åland Islands, and sporadic areas in western and eastern Finland (Figure 3c). Most of central and northern Finland was estimated to have a low probability for *Cs. morsitans* presence, however, excluding sporadic regions with a moderate suitability.

### 3.2. Pogosta Disease Modeling

#### 3.2.1. Predictive Performance

Model performances of the eight modeling approaches and weighted mean ensemble model (biomod2), as well as the generalized linear model (GLM) and random forest (RF) model (VECMAP), are presented in Figure 4. In the biomod2 package, all models provided reasonable estimates (AUC > 0.70) for the distribution of SINV infections resulting in a minimum mean AUC of 0.78 over 50 model runs (0.78 < mean AUC < 0.90). RF and GBM models were the best performing models in biomod2 (0.89 < mean AUC < 0.90, respectively). The weighted mean ensemble model (biomod2), produced by the best-performing model algorithms, yielded the mean AUC of 0.98 with good sensitivity and specificity rates (Figure 4b). In VECMAP, the GLM model resulted in a mean AUC of 0.93 over 100 bootstrap resampling events and a RF model mean AUC of 0.91.

#### 3.2.2. Predictor Contributions to the Distribution of Pogosta Disease Distribution

**Figure A3:**
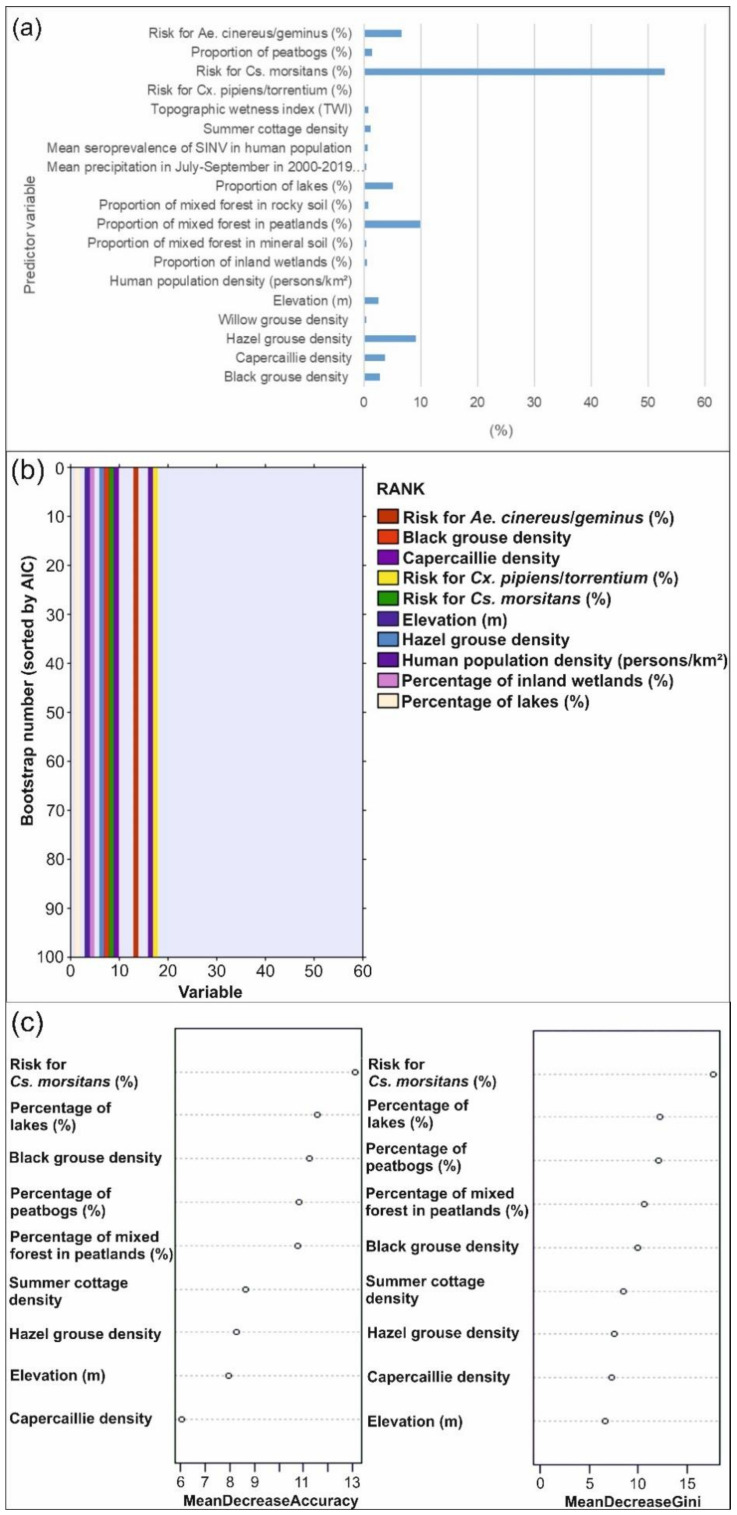
(**a**) Standardized relative contribution of predictors (%) obtained from the weighted mean ensemble model in biomod2, (**b**) variable selection rank order of bootstrap models based on GLM model in VECMAP, and (**c**) variable contribution based on prediction forest in RF model (VECMAP).

The relative contribution of predictors (%) based on the weighted mean ensemble model in biomod2 varied considerably (Figure A3a). The highest relative contribution was provided by the habitat suitability of *Cs. morsitans* (53%), the proportion of mixed forest in peatlands (10%), hazel grouse (9%) density, the habitat suitability of *Ae. cinereus/geminus* (7%), the number of lakes (5%), capercaillie (4%) and black grouse (3%) density per municipality. Based on variable contributions in GLM (VECMAP), all variables were included in the 10 most important variables except for the proportion of mixed forest in peatlands. Furthermore, the habitat suitability for *Cx. pipiens/torrentium*, the proportion of inland wetlands, elevation and human population density were important predictors based on GLM model in VECMAP. The contributions of predictors in the RF model (VECMAP) were mainly consistent in the weighted mean ensemble model and GLM model (VECMAP) (Figure A3b–c).

**Figure 5:**
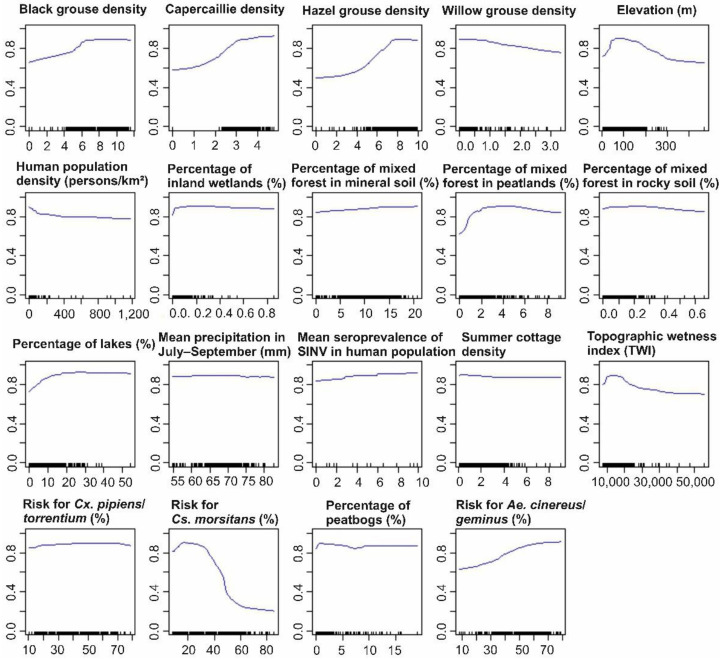
The partial dependency plots for Pogosta disease modeling based on the weighted mean ensemble model produced by biomod2.

Based on the partial dependency plots, high densities of black grouse, capercaillie and hazel grouse indicated high probability of Pogosta disease occurrence (70–98%) (Figure 5). The high willow grouse densities, however, were associated with lower risk for Pogosta disease. A high proportion of mixed forest in peatland, peatbogs and lakes in the municipalities were associated with increased Pogosta disease risk (80–90%). In municipalities at elevations lower than 200 m, the Pogosta disease risk was higher (80–90%), compared to municipalities at higher altitudes. Furthermore, in municipalities at low to high topographic wetness index (TWI) rates, Pogosta disease risk remained high (80–90%). In the municipalities with a high probability of *Ae. cinereus/geminus* occurrence, Pogosta disease risk was also high (80–98%), and remained high also in municipalities with low to high suitability for *Cx. pipiens/torrentium*. In contrast, the disease risk decreased when the habitat suitability for *Cs. morsitans* increased to 50%, whereas in municipalities with low to moderate suitability (0–50%) for *Cs. morsitans*, the Pogosta risk was high (80–90%).

#### 3.2.3. Pogosta Disease Risk Maps

**Figure 6:**
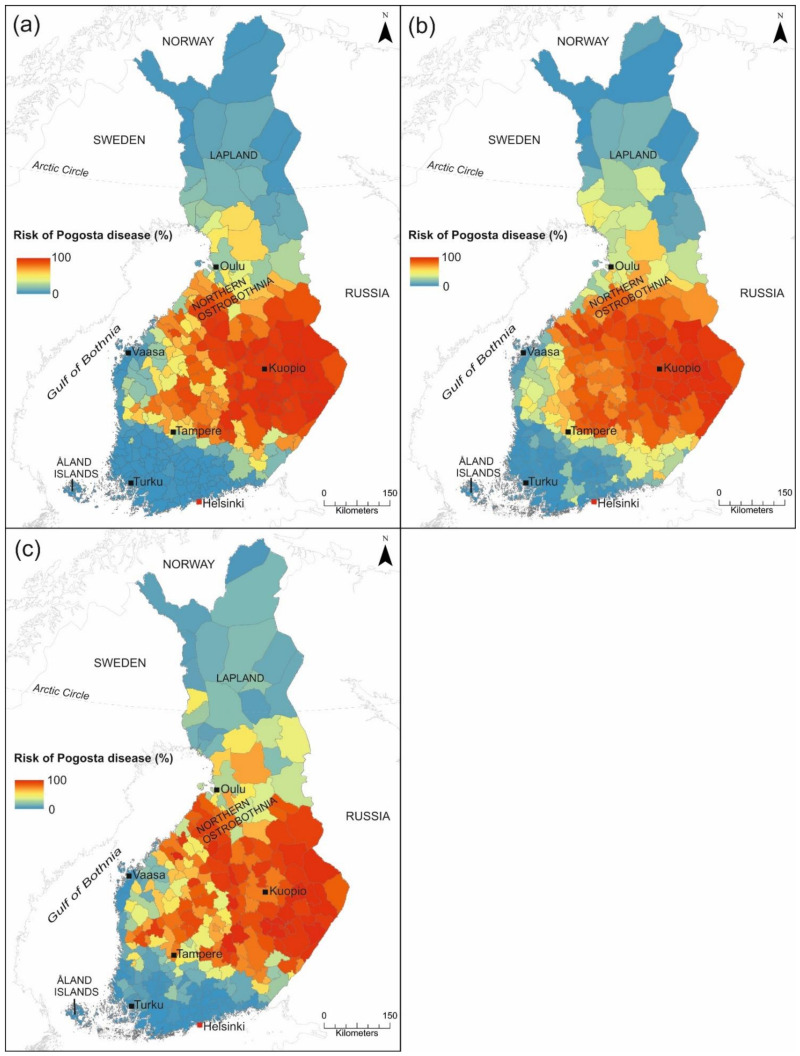
Predicted risk of Pogosta disease in Finland by (**a**) weighted mean ensemble model produced by biomod2, and (**b**) GLM model, and (**c**) RF model produced by VECMAP. The risk is expressed on a scale between 0 (low risk) to 100 (high risk) and visualized with colours ranging from blue (low-risk area) and to red (high-risk area).

The risk map generated from the weighted mean ensemble suggests that a high risk (70–100%) for Pogosta disease occurs in municipalities located in eastern and central Finland, but also in several municipalities along the western coast (Figure 6a). In municipalities bordering high-risk municipalities, the risk of SINV transmission was moderate (30–70%) based on the GLM model (VECMAP, Figure 6b). In contrast, municipalities in northern Lapland, southwestern Finland and the Åland Islands were estimated to be at a low risk (0–20%) for SINV transmission in all predictions (Figure 6a–c). The high-risk areas of Pogosta disease were similar in all prediction maps. Similar results were obtained with biomod2 and VECMAP analyses with the exception that moderate-risk areas in VECMAP predictions were slightly larger to the prediction based on the weighted mean ensemble model in biomod2 (Figure 6a–c).

## 4. Discussion

### 4.1. Validity of the Study

To our knowledge, only a handful of vector-borne disease modeling studies have included suitability data for vectors to predict disease occurrence [6,82]. An ensemble modeling approach was used to predict the potential SINV vectors occurrence and Pogosta disease risk. Ensemble predictions generally yield more accurate estimates over single-model estimates and are widely used to estimate the potential distributions of vectors and vector-borne diseases [83,84]. In VECMAP, both the GLM model and RF models were used to predict Pogosta disease risk. RF models are found to be one of the most accurate model algorithms with high performance in predicting species distributions and are widely used in the field [84,85,86].

Some uncertainty arose from mosquito absences, which were randomly selected from the points where collections were made for a whole-country study [47]. Since collection data covered so many species with differing life histories, any points where potential vectors were absent may not reflect true absences. Among other reasons, absences could be explained by having visited sites when one or more life stages was not active or to be collected or by using collection methods or traps which excluded some species.

There may be also differences in species-specific factors between *Cx. pipiens* and *Cx. torrentium* and between *Aedes cinereus* and *Ae. geminus*, which were pooled in this study. The distribution of *Cx. pipiens* extends to southern Lapland but *Cx. torrentium* is the more dominant of the two species across the whole country. If *Cx. torrentium* truly is the more dominant of the two species in Lapland, then it is unlikely to be involved in bird to human transmission of the virus since it is not reportedly a species which bite humans. Far less is known about the differences between *Ae. cinereus* and *Ae. geminus*, either for biting preferences, or for other behavioral traits. Based on the mosquito collections, *Ae. geminus* is by far the more dominant species of the two across the whole country [47]. Of all the species that are included in the modelling experiment, *Ae. cinereus/geminus* are the most common and voracious biters around the whole country. No experiments have sought to determine if one or the other species is more of a human biter than the other. However, based on the general biting habits of true *Aedes* (12 species), is can be assumed that they would both be aggressive human biters, and as such they would both be involved in the virus transmission. Furthermore, using presence-absence data instead of mosquito abundance data loses information on the relative suitability of habitats when all presences are treated as equal, regardless of the abundance of the individuals that the habitat supports [87]. Pogosta disease patient data [22] are documented by the municipality of residence and may not reflect the actual municipality where patients were infected. Data is also documented based on the date of sample collection rather than the onset of symptoms, which may indicate that there is a time lag of 2–3 weeks to serological diagnosis. Disease awareness among physicians has played a significant role in whether Pogosta disease is diagnosed with serological evidence. As with any infectious diseases with a heterogeneous clinical presentation, it is likely that milder cases or patients that did not experience symptoms did not seek care, and hence would not have been to the NIDR. However, the proportion of unreported cases should not differ regionally. High-resolution data was utilized in the potential vector models, but as Pogosta disease data was available at the municipality level, results were obtained at the same resolution, discarding some of this high-resolution data. We note that other influential variables not considered in this study may exist, such as the occurrence of migratory birds (e.g., passerines), which are known to be associated with SINV infections [8,23,26,88]. However, the number of candidate species of birds potentially involved is too large for all to be included in these models, and a general index of bird abundance may be too nonspecific. In addition, species distribution models (SDMs) of SINV vectors could benefit from micro-climate data or North-Atlantic Oscillation (NAO) index and wind climate [89]. Micro-climate data (spatial resolution < 50 m) better represents thermal and moisture conditions than coarse-scale gridded climate data (≥1 km^2^) [90], but producing microclimate data is computationally intensive and thus it is not yet feasible to apply in SDMs at the municipality scale. The North-Atlantic Oscillation (NAO) index captures the wide spectrum of conditions related to precipitation (water and snow), winds and temperature. In our future studies, we aim to include migratory bird data, the NAO index and future climate data in order to produce more accurate models to predict the occurrence of SINV infections under changing climate conditions.

### 4.2. Influential Variables

Consistent with previous research, environmental and climatic variables were important determinants of SINV vector occurrence. In particular, locations with high mean temperatures in June–August during the studied period, rich vegetation and a long growing season positively influenced the occurrence of *Ae. cinereus/geminus* (Figure A2a). *Aedes cinereus* larvae are known to need a temperature of 12–13 °C to hatch and 14–15 °C to develop, the optimum temperature being 24–25 °C [91]. *Aedes cinereus* is also an acidophilic mosquito, most often found in acido-oligotrophic habitats [34]. Based on our results, short distances to coniferous and mixed forest, low wind speed and low solar radiation in May–September were also suitable habitat conditions for *Ae. cinereus/geminus*. *Aedes cinereus* larvae mostly occur in semi-permanent, partly shaded pools of flood plains, in sedge marshes or bogs, at the edges of lakes covered by emergent vegetation, and in woodland pools [34]. Our study suggests that *Cx. pipiens/torrentium* favour locations with high water vapor pressure, high land surface temperatures in June–August during the studied period, low wind speed and barren vegetation (Figure A2b). *Culex pipiens/torrentium* are widely distributed and able to survive in various habitats, including natural unpolluted and urban polluted habitats close to humans [34,92]. We also found that high precipitation in March–June, high solar radiation in May–September and a long growing season were associated with higher *Cs. morsitans* occurrence (Figure A2c). *Culiseta morsitans* deposit their eggs during early summer in the moist substrate above the residual water level [34,93]. Furthermore, locations with moderate precipitation in July–September and October–February, and with short distances to mixed or coniferous forests, had suitable conditions for *Cs. morsitans* to be present. Suitable sites for *Cs. morsitans* are known to occur in both shaded and open habitats in swampy woodlands and temporary water bodies in forests [34,94].

Our study demonstrates the combined effects of vector species, host species and environmental factors to explain the occurrence of SINV infections. We found that in municipalities with a high probability of *Ae. cinereus/geminus* to occur, the risk for SINV infections was also high. To date, most SINV strains recovered by Swedish studies have been isolated from *Cx. pipiens*, *Cx. torrentium* and *Cs. morsitans* [8,28]. A recent study by Lundström et al. (2019) suggests that the increased prevalence of SINV-I, especially in *Ae. cinereus* and *Cx. pipiens/torrentium*, is a major cause of recent SINV outbreaks in Northern Europe. Our models suggested that the habitat suitability for *Cs. morsitans* negatively influenced the risk of SINV infections. This observation somewhat contradicts the notion that the presence of *Cs. morsitans* is linked to SINV transmission elsewhere in Northern Europe [8,28]. The role of *Cs. morsitans* in SINV transmission has not yet been studied in Finland, but would benefit from more mosquito collection data to boost predictions of presence. The negative relationship may also be in part due to correlation with unobserved variables or due to multicollinearity among predictors.

We found that high densities of hazel grouse, capercaillie and black grouse positively influenced the occurrence of SINV infections, with very similar response functions, indicating the role of resident grouse in the epidemiology of SINV in humans. On the contrary, we found that high willow grouse density was not associated with high Pogosta disease risk as with other resident grouse. Historically the distribution of willow grouse extended from southern Finland to Lapland, but as a result of population decline, the majority of the remaining willow grouse population is nowadays restricted to Lapland [95,96]. Outbreaks of Pogosta disease have previously been reported to follow a 7-year cycle in Finland [21], and were thought to be influenced by the resident grouse populations that also show 6–7-year cycles [97]. Based on the Pogosta disease cases during recent decades (Figure 1a), distinct epidemic cycles are no longer observed. This might be due, in part, to a reduction in the Finnish grouse populations, which were at a record low in 2009, and subsequently reached similar low values during the summers of 2016–2017. Since 2018, however, the population has shown some signs of recovery [23,96]. We also found that a high proportion of mixed forest in peatland, peatbogs, inland wetlands and lakes was associated with increased Pogosta disease risk. These findings that the natural foci of SINV infections mainly occur in wetland ecosystems of diverse biomes, including lowland forested wetlands and humid forests composed of deciduous and coniferous trees, are consistent with previous research from other European locations [26,98].

### 4.3. The Suitability for Potential SINV Vectors and the Risk of Pogosta Disease in Finland

The modeling results suggest that suitable habitats for *Ae. cinereus/geminus* and *Cx. pipiens/torrentium* occur throughout Finland demonstrating their widespread distribution in Europe including Sweden, Finland´s neighboring country (Figure 3a,b) [34,99]. In contrast, suitable habitats for *Cs. morsitans* occurred mainly in southern Finland including sporadic areas in western and eastern Finland (Figure 3c). In part this will be due to the relatively low number of collections made in these locations, and will be compounded by the collections frequently being made at unsuitable times of the year to obtain these species, or by including absence points in the dataset which were made at times when these species were inactive. However, *Cs. morsitans* is found to be species whose distribution ranges from southern Scandinavia to Northern Africa and, based on a Swedish study a majority of *Cs. Morsitans*, observations were documented in the same latitude where their suitability was highest in Finland [99].

Our study results suggest that the highest risk for SINV infections occurs in municipalities located in central, eastern, and western Finland, which is mainly consistent with previous findings about the incidence of Pogosta disease [21,23,25]. However, when comparing the prediction maps (Figure 6a–c) to the Pogosta disease incidence map 2000–2019 (Figure 1b), several differences are evident. Even though a general trend of geographic distribution of high-incidence municipalities was similar to high-risk municipalities, the geographical extent of high-risk municipalities was much wider on the prediction maps produced in this study (Figure 6a–c). Moderate-risk areas extended from southern Lapland to southern Finland, excluding the southern coast. In comparison with the incidence map, the largest differences occurred in western Finland, southern Lapland and Northern Ostrobothnia, where the risk was either high or moderate in several municipalities based on the prediction maps. This is an important detail when determining area-specific recommendations and delivering information to health care personnel to raise awareness of the disease among physicians. The locations with the highest environmental suitability for *Ae. cinereus/geminus* and *Cx. pipiens/torrentium* overlap in geographical range with the municipalities at high risk for SINV infections. In municipalities neighboring high-risk municipalities, the risk of SINV transmission was moderate. We note that northern Lapland, southwestern Finland and the Åland archipelago were estimated to be low-risk areas for SINV transmission. These areas in Finland are also the most extreme ends in terms of wind speed, depth of snow cover or cold air temperatures, and experience less severe heat extremes compared to elsewhere in Finland where climate change impacts are increasing. In northern Lapland, low temperatures and a long winter may halt viral replication and restrict vector populations, which may influence the low probability of Pogosta disease occurrence [100].

## 5. Conclusions

Despite the wide distribution of SINV in the Old World, the reasons for such a distinct geographical region and high numbers of cases in Finland have remained elusive. Our results provide new evidence for the joint influence of vectors, host species and environmental factors in shaping the pattern of SINV infections in Finland. Environmentally suitable areas were identified for the potential SINV vectors *Ae. cinereus/geminus*, *Cx. pipiens/torrentium* and *Cs. morsitans*. Municipalities with an increased risk of Pogosta disease were characterized by high environmental suitability for *Ae. cinereus/geminus*; high densities of black grouse, capercaillie and hazel grouse; a high proportion of mixed forest in peatlands; and a high number of lakes. The risk of transmission was predicted to be greatest in eastern and central Finland, and in several municipalities in western Finland, excluding the coastal areas. Future studies predicting the occurrence of Pogosta disease in Finland should also include the temporal dimension, focusing on the occurrence of potential SINV vectors under different scenarios of land use and climate change, as well as the population dynamics of both host and vector species.

## Figures and Tables

**Table 1:** Description and source of all predictor data.

Data Source	Data Layer(s)	Modifications	Year	Spatial Resolution	References
FMI	Wind speed 50 years return interval (m/s)	Calculated mean wind speed 50 y interval per municipality.	1979–2015	20 m	[50]
FMI	Mean monthly air temperature (°C)	Calculated mean monthly temperature per municipality in 2000–2019 in July–September, October–February and March–June. Air temperature measurement height was 2 m (FMI).	2000–2019	1000 m	[51]
FMI	Mean monthly precipitation (mm)	Calculated mean monthly precipitation per municipality in 2000–2019 in July–September, October–February and March–June.	2000–2019	1000 m	[51]
FMI	Mean monthly snow depth (cm)	Calculated mean monthly snow depth per municipality in 2000–2019 in October–November, December–February and March–April.	2000–2019	1000 m	[51]
FMI	Mean precipitation during growing season (mm)	Calculated mean precipitation during growing season per municipality.	averages for 1981–2010	1000 m	[52]
FMI	Mean heat summation during growing season (°C day)	Calculated mean heat summation during growing season per municipality.	averages for 1981–2010	1000 m	[52]
FMI	Growing season length (GLS) (day)	Calculated growing season length (GLS) per municipality.	averages for 1981–2010	1000 m	[52]
LUKE	Density (individuals/km^2^) of willow grouse (*Lagopus lagopus*), black grouse (*Lyrurus tetrix*), capercaillie (*Tetrao urogallus*) and hazel grouse (*Tetrastes bonasia*)	Average annual densities at a 100 km radius from the municipality center, further averaged over the years. Based on wildlife triangle census.	2000–2019	Municipality	[49]
SYKE, EEA, EU/Copernicus programme	CORINE land cover 2018	Euclidean distances to selected land cover types from mosquito species PA point were calculated in ArcGIS. Proportion (%) of chosen land cover types were derived by calculating percentage of each land cover type for municipality.	2018	20 m	[53]
Statistics Finland	Human population density (persons/km^2^)	Calculated as sum per municipality.	2019	1000 m	[54]
Statistics Finland	Summer cottage density (cottages/km^2^)	Calculated in ArcGIS in order to present summer cottages per area (km^2^) of municipality.	2019	Municipality	[54]
Kurkela et al. 2008	Mean seroprevalence of SINV in human population in Finland	Seroprevalence rate was taken from the hospital district in which municipality belongs to.	1999–2003	Hospital districts	[23]
NLS of Finland	Topographic wetness index (TWI)	Calculated mean TWI per municipality.	2016	16 × 16 m	[55]
NLS of Finland	Digital elevation model (m)	Calculated mean elevation per municipality.	2019	10 × 10 m	[56]
WorldClim- Global climate data	Solar radiation (kJ m^−2^ day^−1^)	Calculated mean solar radiation in May–September.	averages for 1980–2000	~1000 m	[57]
WorldClim- Global climate data	Water vapor pressure (kPa)	Calculated mean water vapor pressure in May–September.	averages for 1980–2000	~1000 m	[57]
NASA Earthdata	Normalized Difference Vegetation Index (NDVI) (MOD13A3)	Calculated mean NDVI in June 2000–2019 per municipality.	in June, 2000–2019	1000 m	[58]
NASA Earthdata	Land surface temperature (°C) (MOD11C3)	Calculated mean monthly LST per municipality in 2000–2019 April–May, June–August and September–October	2000–2019	5600 m	[59]
Lorna Culverwell, Jenny Hesson	The distribution data of *Cs. morsitans*, *Cx.pipiens*/*torrentium* and *Ae. cinereus*/*geminus*	The occurrence data of *Cx. pipiens* and *Cx. torrentium*, and *Ae. cinereus* and *Ae. geminus*, were unified to *Cx. pipiens/torrentium* and *Ae. cinereus/geminus* due to the lack of reliable identification methods to distinguish them to either of the species.	2009–2018	Location (Longitude, Latitude)	[46,47]
THL, NIDR	Patient Pogosta disease data	Pogosta disease average incidence per 1000 inhabitants during 2000–2019 per municipality was calculated in ArcGIS.	2000–2019	Municipality	[22]

FMI = Finnish Meteorological Institute; LUKE = Natural Resources Institute Finland; SYKE = Finnish Environment Institute; EEA = European Environment Agency; EU = European Union; THL = Finnish Institute for Health and Welfare; NIDR = National Infectious Disease Register.

**Table 2:** Final environmental and other predictors used in (a) potential SINV vector species modeling, and (b) Pogosta disease modeling with value ranges.

(a)	*Ae. cinereus/geminus*			*Cx. pipiens/torrentium*			*Cs. morsitans*		
Predictor	Min.	Max.	Avg.	Min.	Max.	Avg.	Min.	Max.	Avg.
Wind speed 50 years interval	10.5	41.3	14.4	10.5	14.6	14.6	10.5	36.9	14.6
Topographic wetness index (TWI)	4269	65,535	16,708	4617	65,535	15,261	4617	65,535	15,261
Mean snow depth in October–November 2009–2019	x	x	x	0.2	12.4	2.3	0.2	12.6	2.3
Mean precipitation in October–February 2009–2019	26.3	68.8	47.4	26.3	49	49	26.8	68.8	49
Mean precipitation in March–June 2009–2019	33.5	54.7	42.2	33.5	54.3	41.9	33.5	54.3	41.9
Mean precipitation in July–September 2009–2019	55.8	85.3	67.5	54.7	84.7	67.1	56.6	84.7	67.1
Mean normalized difference vegetation index (NDVI) in June 2009–2019	0.4	0.8	0.7	0.4	0.8	0.7	0.5	0.8	0.7
Mean water vapor pressure in May–September 2009–2019	x	x	x	0.8	1.21	1.04	x	x	x
Human population density	0	4281	141.9	0	4281	73.4	0	4281	73.4
Euclidean distance to water courses	0	19,194.3	2485.6	0	20,634.4	2884.2	0	19,194.3	2884.2
Euclidean distance to water bodies	0	4628.9	665.8	0	4628.9	757.1	0	4628.9	757.1
Euclidean distance to peatbogs	0	4891.8	1021.5	20	4303.6	1093.1	0	4891.8	1093.1
Euclidean distance to inland marshes	0	12,041.4	2155.2	0	12,041.4	2182.8	44.7	12,041.4	2182.8
Euclidean distance to coniferous forest	0	2272.7	98.2	0	3217.6	76.3	0	570.1	76.3
Euclidean distance to broad-leaved forest	0	2062.4	201.6	0	906.9	170.4	0	1063.2	170.4
Euclidean distance to mixed forest	0	1724.1	136.6	0	3265	119.3	0	1668.8	119.3
Euclidean distance to transitional woodland/shrub	0	1073.6	141.9	0	1073.6	135.3	0	738.2	135.3
Mean land surface temperature in June–August 2009–2019	13.3	24	18.4	12.4	23.9	18.7	14.7	22.6	18.7
Mean precipitation during growth season 1981–2010	93.1	190.3	157.8	184.1	378.5	326.5	101.9	190.3	162.6
Mean solar radiation in May–September	13,718	17,526	15,460	x	x	x	13,639	17,526	15,662
Elevation	0.1	116.5	564.6	x	x	x	x	x	x
**(b)**	**Pogosta disease**								
**Predictor**	**Min.**	**Max.**	**Avg.**						
Black grouse density	0	11.6	6.9						
Capercaillie density	0	4.9	3.2						
Hazel grouse density	0	10	7.3						
Willow grouse density	0	3.3	0.3						
Elevation (m)	4.9	471.3	94.2						
Human population density (persons/km^2^)	0	1177	36.7						
Percentage of inland wetlands (%)	0	0.9	0.1						
Percentage of mixed forest in mineral soil (%)	0	20.9	10.4						
Percentage of mixed forest in peatlands (%)	0	9.3	2						
Percentage of mixed forest in rocky soil (%)	0	0.7	0.1						
Percentage of lakes (%)	0	54.4	8.4						
Mean precipitation in July–September in 2000–2019 (mm)	54.8	82.8	68.2						
Mean seroprevalence of SINV in human population	0	9.9	3.5						
Summer cottage density	0.1	9.1	1.8						
Topographic wetness index (TWI)	7751	65,535	17,062						
Suitability for *Cx. pipiens/torrentium* (%)	10.6	78.7	41.3						
Risk for *Cs. morsitans* (%)	8.3	85.9	36.2						
Percentage of peatbogs (%)	0	19.1	2.1						
Risk for *Ae. cinereus/geminus* (%)	8.5	78.7	50.9						

x = Predictor not included in the final dataset.

## Data Availability

The data presented in this study are available in the article.
